# Seven Sycoryctine Fig Wasp Species (Chalcidoidea: Pteromalidae) Associated with Dioecious *Ficus hirta* Inhabiting South China and Southeast Asia

**DOI:** 10.3390/biology11060801

**Published:** 2022-05-24

**Authors:** Da-Mien Wong, Songle Fan, Hui Yu

**Affiliations:** 1 Southern Marine Science and Engineering Guangdong Laboratory (Guangzhou), Guangzhou 511458, China; dmwong87@gmail.com (D.-M.W.); fansongle@scbg.ac.cn (S.F.); 2 Key Laboratory of Plant Resource Conservation and Sustainable Utilization, South China Botanical Garden, Chinese Academy of Sciences, Guangzhou 510650, China; 3 Guangdong Provincial Key Laboratory of Digital Botanical Garden, South China Botanical Garden, Chinese Academy of Sciences, Guangzhou 510650, China

**Keywords:** inquilines, Indomalaya, non-pollinating fig wasps, parasitoids, Sycoryctinae

## Abstract

**Simple Summary:**

The non-pollinating fig wasps are essential components of fig wasp communities, negatively impacting mutualism. However, this group of fig wasps has received less taxonomic attention than pollinating fig wasps. This study presents seven new non-pollinating fig wasp species associated with *Ficus hirta* fig trees inhabiting South China and Southeast Asia. The presence of a long ovipositor sheath characterizes this group of fig wasps. An identification key is provided to distinguish between them, and the relationships with their host fig trees are discussed. The type specimens and examined materials are deposited in the South China Botanical Garden, Chinese Academy of Sciences, China.

**Abstract:**

Even though non-pollinating fig wasps are essential components in tropical and subtropical habitats, yet they are poorly described in the Oriental communities. This study presented seven new sycoryctine fig wasp species associated with *Ficus hirta* fig trees inhabiting South China and Southeast Asia. These new sycoryctine species belong to the genera *Philotrypesis*, *Sycoryctes*, and *Sycoscapter*. They can be easily distinguished by their adaptive morphologies such as face sculpture, body-color, and ovipositors. An identification key is provided to differentiate between them, and the relationships with their host fig trees are also discussed. The holotypes and paratypes are both deposited in the South China Botanical Garden, Chinese Academy of Sciences, China.

## 1. Introduction

Fig wasps are exciting for ecological and evolutionary studies, particularly their adaptive morphologies and co-speciation with their host fig trees [1,2]. Despite most of the early accounts of fig wasp focusing on pollinating species, there can be up to 30 diverse non-pollinating species associated with a single host fig tree [3]. Although these tiny hymenopterans could play an important role in tropical- and sub-tropical ecology, the non-pollinating fig wasps are still poorly described in the Oriental realm [4].

Sycoryctine fig wasps (Chalcidoidea: Pteromalidae) are non-pollinators of fig trees. The taxonomy of sycoryctines has changed over the past ten years, and their phylogeny is currently more defined due to the advancement of molecular techniques [5,6]. These fig wasps belong to the subfamily Sycoryctinae and are highly diversified and geographically widespread [5]. Sycoryctines are associated with all six subgenera and at least 15 sections in *Ficus* [7]. Surprisingly, an estimated 826 species (2.7 species per *Ficus*) are waiting to be discovered in the Old World [5]. Two sycoryctine species have been described recently in Taiwan [8] and India [9], and hopefully more Oriental sycoryctines are going to be described.

Sycoryctinae is currently divided into four tribes: (1) Apocryptini, comprising two genera: *Apocrypta* Coquerel, and *Bouceka* Kocak and Kemal; (2) Critogastrini, comprising only one genus: *Critogaster* Mayr; (3) Philotrypesini, comprising four genera: *Dobunabaa* Boucek, *Philoverdance* Priyadarsanan, *Philotrypesis* Forster, and *Watshamiella* Wiebes; and (4) Sycoryctini, comprising seven genera: *Adiyodiella* Priyadarsanan, *Arachonia* Joseph, *Parasycobia* Abdurahiman and Joseph, *Sycorycteridea* Abdurahiman and Joseph, *Sycoryctes* Mayr, *Sycoscapter* Saunders, and *Sycoscapteridea* Ashmead [5,10]. The sycoryctine fig wasps are considered host specificity conservatisms which originated about 49–64 million years ago [5]. The sycoryctine species are also believed to impact the fig–fig wasp mutualism significantly [11]. Female sycoryctines have remarkable long ovipositors that can penetrate the fig wall. They attack flowers containing other fig wasp larvae by consuming the host larvae or starving them by feeding on endosperm [12].

Their complicated trophic relationships make sycoryctines the ideal study species for the population dynamics in tropical and sub-tropical habitats [13]. A recent molecular study showed eight allopatric sycoryctine species associated with *Ficus hirta* Vahl in South China and Southeast Asia. The marked barcoding gaps ranged from 7.2% to 15.7% for the Cytb gene sequence in the same genus [14]. However, the morphological traits of these species remain unknown. This study compared their morphology and reported seven new species belonging to the genera *Philotrypesis*, *Sycoryctes*, and *Sycoscapter*. An identification key is provided to distinguish between them, and the relationships with their host fig trees are discussed.

Like the non-pollinating fig wasps, sycoryctines often receive less research attention in the symbiosis of figs and fig wasps. Hence, this study is concerned with the taxonomy of the geographically widespread sycoryctine fig wasps associated with *F. hirta*. Our research and results provide new insights into the morphology and adaptation of these non-pollinating fig wasps. It contributes to our understanding of speciation and biodiversity in the Oriental fig–fig wasp communities.

## 2. Materials and Methods

The specimens of sycoryctine fig wasps associated with *F. hirta* were collected in 23 sampling sites for *Philotrypesis*, 15 sites for *Sycoscapter*, and two sites for *Sycoryctes* during the years 2010 to 2018 are distributed from South China to Java (Table 1). The distribution of the species in each genus is allopatric except for two sites with two species in each genus co-occurred, QMS in Thailand and XI in China (Table 1). When figs close to ripening were dissected, the wasps emitted therein were collected. Two to twenty-four wasps in each site with one wasp exited from one natal fig were collected and stored in 75% ethanol. Later, the wasps were dehydrated through an ethanol series (80%, 90%, and 100%) and critical-point dried (LEICA EM CPD300, Leica Microsystems GmbH, Wetzlar, Germany) before being mounted on cards following Noyes (1982) [15]. Each photo was taken using a digital camera connected to a stereomicroscope (LEICA M205 FA, Leica Microsystem GmbH, Wetzlar, Germany). The images were processed using LAS X 3.08.19082 software to create a stacked image with increased focal depth [16]. Physical characteristics were measured using ImageJ 1.8.0_172 software (National Institutes of Health, Bethesda, MD, USA). Specimen measurements were taken with an accuracy of 0.001 mm and rounded to the nearest 0.01 mm.

Specimens were mounted on brass stubs and sputter-coated with gold (LEICA EM ACE600, Leica Microsystem GmbH, Germany) before the observation and photographed using SEM (JEOL JSM-6360LV, JEOL Ltd., Tokyo, Japan). Morphological terminology follows Gibson (1997) and the Hymenoptera Anatomy Ontology (HAO) Portal [17,18]. The holotypes and a group of paratypes are deposited in the Plant Science Center, South China Botanical Garden, Chinese Academy of Sciences (23°10′48″ N; 113°21′8″ E).

## 3. Results

### 3.1. Philotrypesis Forster, 1878

#### 3.1.1. *Philotrypesis* Forster, 1878: 153–187. Type Species: *Philotrypesis Longicaudata* Mayr, 1906

Diagnosis: The female of this genus can be recognized by its lengthened seventh and eighth urotergites and its subquadrate pronotum.

Distribution and host relationships (from www.figweb.org, accessed on 9 July 2021): *Philotrypesis* fig wasp species are known from Afrotropical realm: Eritrea, Guinea, Sierra Leone, South Africa, Zambia, Zimbabwe; Australasian realm: Australia, Indonesia; Nearctic realm: United States; Oriental realm: Indonesia, Japan, Mainland China, Malaysia, Philippines, Sri Lanka, Taiwan, Vietnam; and Palearctic realm: France, Israel, Italy. All described *Philotrypesis* fig wasp species are parasitoids or inquilines of other fig wasps associated with sections *Conosycea*, *Ficus*, *Galoglychia*, *Sycidium*, *Sycocarpus*, and *Urostigma* fig trees. The recorded host fig wasps of *Philotrypesis* included *Blastophaga psenes* Linnaeus, *Ceratosolen dentifer* Wiebes, *Ceratosolen notus* Baker, *Ceratosolen solmsi* Mayr, *Eupristina verticillata* Waterston, *Kradibia brownii* Ashmead, *Kradibia gestroi* Grandi, and *Platyscapa quadraticeps* Mayr.

##### *Philotrypesis guangdongensis* Yu sp. n.

**Figure 1:**
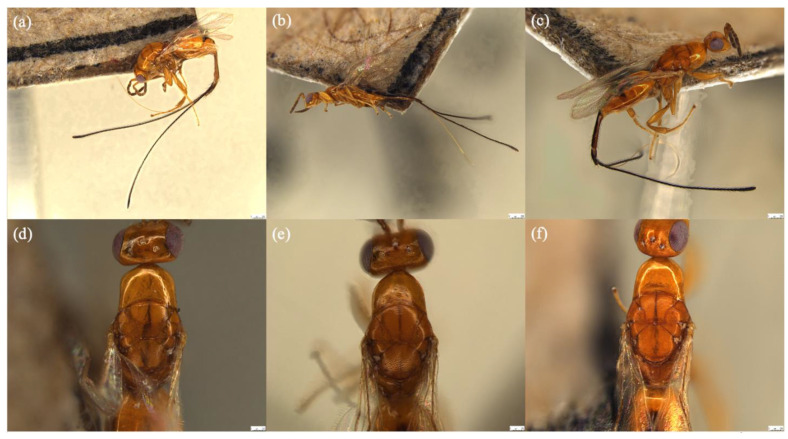
Habitus lateral of (**a**) *Philotrypesis guangdongensis* sp. n.; (**b**) *P. yunnanensis* sp. n.; (**c**) *P. fujianensis* sp. n. Mesosoma, dorsal view of (**d**) *P. guangdongensis*; (**e**) *P. yunnanensis*; (**f**) *P. fujianensis*. Noted that the black band of *P. yunnanensis* on scutellum is indistinct. Scale bars represent 250 μm.

**Figure 2:**
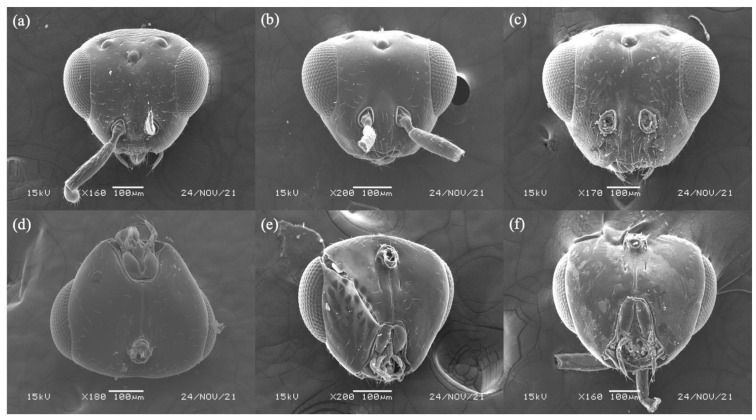
Head, dorsal view of (**a**) *Philotrypesis guangdongensis* sp. n.; (**b**) *P. yunnanensis* sp. n.; (**c**) *P. fujianensis* sp. n. Head, ventral view of (**d**) *P. guangdongensis*; (**e**) *P. yunnanensis*; (**f**) *P. fujianensis*. Noted that the toruli of *P. guangdongensis* are located below the bottom line of compound eyes, and the mouthpart of *P. fujianensis* extended below to the central of compound eyes.

**Figure 3:**
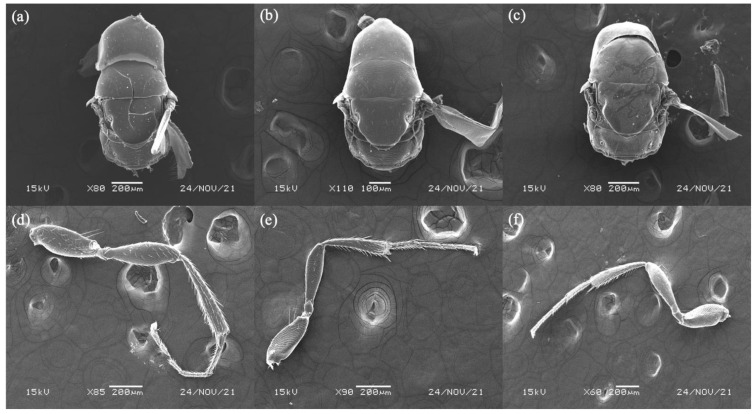
Mesosoma, dorsal view of (**a**) *Philotrypesis guangdongensis* sp. n.; (**b**) *P. yunnanensis* sp. n.; (**c**) *P. fujianensis* sp. n. Hind leg of (**d**) *P. guangdongensis*; (**e**) *P. yunnanensis*; (**f**) *P. fujianensis*.

(Figure 1a,d, Figure 2a,d and Figure 3a,d)

Distribution: China (Guangdong, Guangxi, Hainan provinces, and Hong Kong SAR), Thailand, Vietnam.

Types: Holotype, ♀, CHINA: Guangzhou, 23°10′12.0″ N, 113°22′22.8″ E, 27 November 2015, H. Yu. Paratypes, 4♀, same locality and data as holotype.

Description: Female. Color and Size. Body length 1.9–2.1 mm. Body color yellowish orange. Head and antennae orange. Mesosoma and metasoma usually yellowish orange. Coxae concolorous with mesosoma. Wings hyaline. The 7th and 8th segment in ratio 4:1. Ovipositor sheath length 2.5–3.0 mm.

Head. Width 0.4–0.5 mm. Eye longer than gena. Antenna inserted below the bottom line of compound eye. Toruli apart, distance between toruli larger than diameter of one torulus. Funicular segments slightly longer than wide. Face sculpture smooth. Epistomal margin flattened.

Mesosoma. Length 2.0–2.2 mm. Wing length 1.3–1.5 mm and finely pubescent. Black band on scutellum distinct; black band on mesoscutum bifurcation.

Metasoma. Length 1.8–2.0 mm. Without petiole. Ovipositor sheath length 2× longer than body.

Male. Unknown.

Etymology: Named after the Guangdong province of China.

##### *Philotrypesis yunnanensis* Yu sp. n.

(Figure 1b,e, Figure 2b,e and Figure 3b,e)

Distribution: China (Guizhou and Yunnan Provinces), Thailand.

Types: Holotype, ♀, CHINA: Yunnan, 21°26′49.2″ N, 101°34′04.8″ E, 4 July 2013, H. Yu. Paratypes, 4♀, same locality and data as holotype.

Description: Female. Color and Size. Body length 1.9–2.1 mm. Body color yellowish orange. Head and antennae orange. Mesosoma and metasoma usually yellowish orange. Coxae concolorous with mesosoma. Wings hyaline. The 7th and 8th segment in ratio 4:1. Ovipositor sheath length 2.5–2.9 mm.

Head. Width 0.3–0.4 mm. Eye longer than gena. Antenna inserted below the bottom line of compound eye. Toruli apart, distance between toruli larger than diameter of one torulus. Funicular segments slightly longer than wide. Face sculpture smooth. Epistomal margin flattened.

Mesosoma. Length 2.0–2.2 mm. Wing length 1.3–1.4 mm and finely pubescent. Black band on scutellum indistinct; black band on mesoscutum straight.

Metasoma. Length 1.6–2.0 mm. Without petiole. Ovipositor sheath length 2× longer than body.

Male. Unknown.

Etymology: Named after the Yunnan province of China.

##### *Philotrypesis fujianensis* Yu sp. n.

(Figure 1c,f, Figure 2c,f and Figure 3c,f)

Distribution: China (Fujian, Guangdong, and Jiangxi Provinces).

Types: Holotype, ♀, CHINA: Fujian, 26°39′50.4″ N, 119°32′56.4″ E, 24 January 2016, H. Yu. Paratypes, 4♀, same locality and data as holotype.

Description: Female. Color and Size. Body length 1.9–2.3 mm. Body color yellowish orange. Head and antennae orange. Mesosoma and metasoma usually yellowish orange. Coxae concolorous with mesosoma. Wings hyaline. The 7th and 8th segment in ratio 4:1. Ovipositor sheath length 2.6–2.9 mm.

Head. Width 0.4–0.8 mm. Eye longer than gena. Antenna inserted at the bottom line of compound eye. Toruli apart, distance between toruli larger than diameter of one torulus. Funicular segments slightly longer than wide. Face sculpture smooth. Epistomal margin flattened.

Mesosoma. Length 2.0–2.4 mm. Wing length 1.3–1.5 mm and finely pubescent. Black band on scutellum distinct; black band on mesoscutum straight.

Metasoma. Length 1.8–2.1 mm. Without petiole. Ovipositor sheath length 1.5× longer than body.

Male. Unknown.

Etymology: Named after the Fujian province of China.

### 3.2. Sycoryctes Mayr, 1885

#### 3.2.1. *Sycoryctes* Mayr, 1885: 153–187. Type Species: *Sycoryctes Patellaris* Mayr, 1885

Diagnosis: Stigmal knob not produced downwards. Dorso-apical spine on basitarsus short, not reaching end of second segment.

Distribution and host relationships (from www.figweb.org, accessed on 9 July 2021): *Sycoryctes* fig wasp species are mainly known from the Afrotropical, Australasian, and Oriental realms. All described *Sycoryctes* fig wasp species are parasitoids or inquilines of other fig wasps.

##### *Sycoryctes javaensis* Yu sp. n.

(Figure 4)

Distribution: Indonesia.

Types: Holotype, ♀, INDONESIA: Java, 6°22′04.8″S, 106°49′48.0″E, 3 MAY 2014, H. Yu. Paratypes, 4♀, same locality and data as holotype.

Description: Female. Color and Size. Body length 1.2–1.6 mm. Body color metallic green with brownish reflection. Head and antennae metallic green. Mesosoma and metasoma metallic green. Coxae yellow. Wings hyaline. Ovipositor sheath length 1.6–2.0 mm.

Head. Width 0.2–0.5 mm. Eye longer than gena. Antenna inserted above the bottom line of compound eye. Toruli approach, distance between toruli smaller than diameter of one torulus. Funicular segments slightly longer than wide. Face sculpture smooth. Epistomal margin slightly protruded.

Mesosoma. Length 1.1–1.5 mm. Wing length 1.2–1.4 mm and finely pubescent.

Metasoma. Length 1.0–1.4 mm. Without petiole. Ovipositor sheath length 3× longer than body.

Male. Unknown.

Etymology: Named after the Java Island of Indonesia.

### 3.3. Sycoscapter Saunders, 1883

#### 3.3.1. *Sycoscapter Saunders*, 1883: 29–47. Type Species: *Sycoscapter Insignis Saunders*, 1883

Diagnosis: Stigmal knob not produced downwards. Funicular segments symmetric.

Distribution and host relationships (from www.figweb.org, accessed on 9 July 2021): *Sycoscapter* fig wasp species are mainly known from the Afrotropical, Australasian, and Oriental realms. All described *Sycoscapter* fig wasp species are parasitoids of other fig wasps. The recorded host fig wasps of *Sycoscapter* included *Ceratosolen dentifer* Wiebes, *Eupristina delhiensis* Abdurahiman and Joseph, *Eupristina verticillata* Waterston, and *Kradibia gestroi* Grandi.

##### *Sycoscapter chinensis* Yu sp. n.

(Figure 5a,b and Figure 6a)

Distribution: China (Guangdong, Guangxi, Guizhou, Hainan, Yunnan Provinces, and Hong Kong SAR).

Types: Holotype, ♀, CHINA: Guangzhou, 23°10′12.0″ N, 113°22′22.8″ E, 27 November 2015, H. Yu. Paratypes, 4♀, same locality and data as holotype.

Description: Female. Color and Size. Body length 1.6–2.0 mm. Body color metallic green. Head and antennae metallic green. Mesosoma and metasoma metallic green. Coxae yellow. Wings hyaline. Ovipositor sheath length 1.6–2.0 mm.

Head. Width 0.3–0.5 mm. Eye longer than gena. Antenna inserted at the bottom line of compound eye. Toruli approach, distance between toruli smaller than diameter of one torulus. Funicular segments slightly longer than wide. Face sculpture smooth. Epistomal margin slightly protruded.

Mesosoma. Length 1.2–1.7 mm. Wing length 1.3–1.7 mm and finely pubescent.

Metasoma. Length 1.2–1.6 mm. Without petiole. Ovipositor sheath length 3.5× longer than body.

Male. Unknown.

Etymology: Named after China.

##### *Sycoscapter thaiensis* Yu sp. n.

(Figure 5b,e and Figure 6b)

Distribution: Thailand.

Types: Holotype, ♀, THAILAND: Mueang Chiang Mai, 18°48′32.4″ N, 98°54′50.4″ E, 3 July 2014, H. Yu. Paratypes, 4♀, same locality and data as holotype.

Description: Female. Color and Size. Body length 1.7–2.1 mm. Body color metallic green with brownish reflection. Head and antennae metallic green. Mesosoma and metasoma metallic green. Coxae yellow. Wings hyaline. Ovipositor sheath length 1.7–2.0 mm.

Head. Width 0.4–0.6 mm. Eye longer than gena. Antenna inserted at the bottom line of compound eye. Toruli approach, distance between toruli smaller than diameter of one torulus. Funicular segments slightly longer than wide. Face sculpture deep. Epistomal margin protruded.

Mesosoma. Length 1.3–1.7 mm. Wing length 1.3–1.5 mm and finely pubescent.

Metasoma. Length 1.4–1.6 mm. Without petiole. Ovipositor sheath length 3.5× longer than body.

Male. Unknown.

Etymology: Named after Thailand.

##### *Sycoscapter singaporensis* Yu sp. n.

(Figure 5c,f and Figure 6c)

Distribution: Singapore.

Types: Holotype, ♀, SINGAPORE: Tanglin, 1°18′43.2″ N, 103°48′57.6″ E, 19 August 2013, H. Yu. Paratypes, 4♀, same locality and data as holotype.

Description: Female. Color and Size. Body length 1.8–2.0 mm. Body color metallic green with blue reflection. Head and antennae metallic green. Mesosoma and metasoma metallic green. Coxae yellow. Wings hyaline. Ovipositor sheath length 1.8–2.2 mm.

Head. Width 0.4–0.6 mm. Eye longer than gena. Antenna inserted at the bottom line of compound eye. Toruli approach, distance between toruli smaller than diameter of one torulus. Funicular segments slightly longer than wide. Face sculpture deep. Epistomal margin protruded.

Mesosoma. Length 1.4–1.6 mm. Wing length 1.3–1.5 mm and finely pubescent.

Metasoma. Length 1.4–1.5 mm. Without petiole. Ovipositor sheath length 4× longer than body.

Male. Unknown.

Etymology: Named after Singapore.

### 3.4. Diagnoses of Female Sycoryctine Species Associated with Ficus Hirta

The female of *Philotrypesis guangdongensis* is morphologically similar to *P. yunnanensis* and *P. fujianensis*; however, its toruli located slightly below the bottom line of compound eyes. The mouthpart of *P. fujianensis* is longer than *F. guangdongensis* and *F. yunnanensis* and it extended just below the central of the compound eyes. The black band of *P. yunnanensis* on scutellum is indistinct compared to *P. guangdongensis* and *P. fujianensis*, *P. yunnanensis* also has a non-bifurcated line on its mesoscutum.

Both the females belong to the genus *Sycoryctes* and *Sycoscapter* have metallic green body color and a relatively long ovispositor; however, the knob of *Sycoryctes* on stigmal vein does not produce downward. *Sycoscapter* *chinensis* does not has deep face sculpture and an acute epistomal margin projection compared to *S. thaiensis* and *S. singaporensis*.

### 3.5. Key to Female Sycoryctine Species Associated with Ficus Hirta

**Figure 4:**
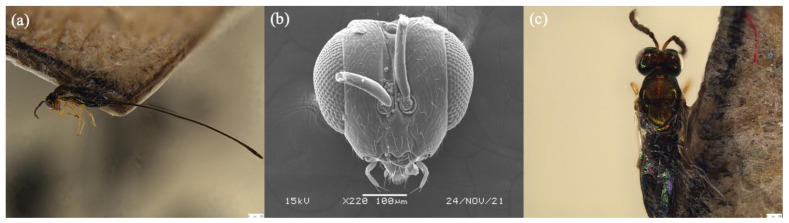
*Sycoryctes javaensis* sp. n. in (**a**) habitus lateral view; (**b**) head dorsal view; and (**c**) mesosoma dorsal view. Scale bars on the stacked images are 250 μm.

**Figure 5:**
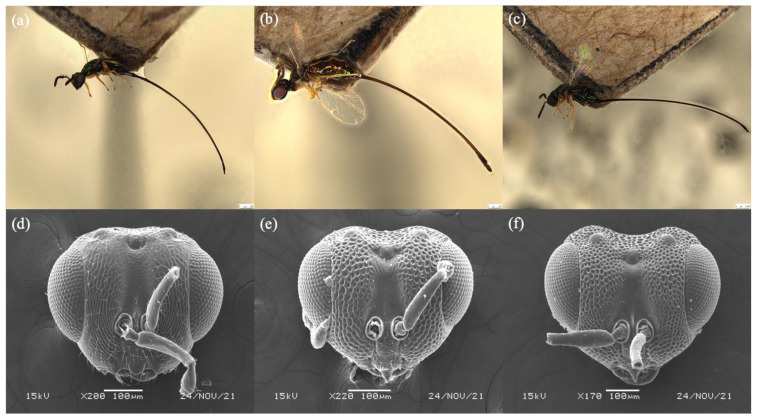
Habitus lateral view of (**a**) *Sycoscapter chinensis* sp. n.; (**b**) *S. thaiensis* sp. n.; (**c**) *S. singaporensis* sp. n. Head, dorsal of (**d**) *S. chinensis*; (**e**) *S. thaiensis*; (**f**) *S. singaporensis*. Noted that *S. thaiensis* and *S. singaporensis* have deep face sculptures. Scale bars on the stacked images are 250 μm.

**Figure 6:**
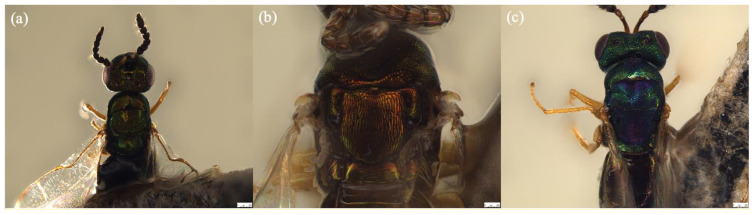
Mesosoma, dorsal view of (**a**) *Sycoscapter chinensis* sp. n.; (**b**) *S. thaiensis* sp. n.; and (**c**) *S. singaporensis* sp. n. Noted that *S. singaporensis* has body color of metallic green with blue reflection. Scale bars represent 250 μm.

1a. Toruli apart; gastral tail consists of two last tergites, ovipositor and its sheaths; stigmal vein without knob; body non-metallic gloss (*Genus Philotrypesis* Forster) …………. 2
1b. Toruli approach and located above the bottom line of compound eyes; gastral tail consists of a last tergites, ovipositor and its sheaths; stigmal vein with a knob; body with metallic gloss ………………………………………………………………………………………………4
2a. Black band on scutellum indistinct (Figure 1b) …………………. *P. yunnanensis* sp. n.
2b. Black band on scutellum distinct ……………………………………………………… 3
3a. Toruli located below the bottom line of compound eyes (Figure 2a); black band on mesoscutum bifurcation (Figure 1d) …………………………… *P. guangdongensis* sp. n.
3b. Mouthpart extended below the central of compound eyes (Figure 2f); black band on mesoscutum does not bifurcate ………………………………………… *P. fujianensis* sp. n.
4a. Epistomal margin without acute projection (Figure 4b); knob on stigmal vein does not elongate; wing pilosity strongly reduced ……………………. *Sycoryctes javaensis* sp. n.
4b. Epistomal margin with an acute projection; fore wing with some long robust hairs below the marginal vein (Genus *Sycoscapter* Saunders) ……………………………………………………………………………………………… 5
5a. Face without deep sculpture (Figure 5d) ……………………………… *S. chinensis* sp. n.
5b. Face with deep sculpture ………………………………………………………………. 6
6a. Metallic green body color with brownish reflection (Figure 6b) …… *S. thaiensis* sp. n.
6b. Metallic green body color with blue reflection (Figure 6c) ……. *S. singaporensis* sp. n.

## 4. Discussion

This study confirmed that the dioecious *F. hirta* inhabiting Southeast Asia is associated with at least seven morphologically distinct sycoryctine fig wasp species. The seven sycoryctine species associated with *F. hirta* can be distinguished morphologically by antennae, epistomal margin, face sculpture, body-color, and ovipositors. This study shows that the number of non-pollinating species on a dioecious fig tree across many geographical areas is higher than previously thought. The limited number of non-pollinating species is either due to the less sampling effort or may be due to the low dispersal ability of fig wasps in the dioecious fig community, which may promote the diversification of these sycoryctine fig wasps [14].

*F. hirta* is a shrub widely distributed in the tropics and subtropics from Java in the south to China in the north and westwards into northeast India [19]. It was initially thought to be symbiosis with one pollinating species and two non-pollinating species [20]. However, through our extensive geographical sampling and molecular sequencing analysis, the non-pollinating fig wasps in the genus of *Philotrypesis* and *Sycoscapter*, which have initially been considered one species, are divided into four and three species, respectively [14]. These non-pollinators are mainly allopatric distributed. The differences in barcode gaps among them in the same genus are no more than 15.7%. Compared with the same genus in other fig species [5], these species are closely related. Some cases have been found in other broadly distributed fig species, such as *F. pumila* [21], *F. racemosa* [22], and *F. septica* [23]. Those results suggest that fig wasps are more likely to differentiate into new species due to their relatively short generation time than their host figs.

Although we have found more related species using molecular sequencing in both pollinating and non-pollinating fig wasps across wide geographical distribution within the same fig species [14,24], our identification of these non-pollinating fig wasps showed that they showed significant differentiation in morphology. Non-pollinating fig wasps of *F. hirta* lay eggs by inserting their long ovipositor through the fig wall. The fig wall within a single fig species varied largely under different environmental conditions [25]. For example, the fig wall thickness of *F. hirta* at the northern limit of China is thicker than that of south China. Accordingly, the ovipositor length of *Philotrypesis fujianensis* distributed there is also significantly longer.

Exploring the speciation or host switching in the conservative sycoryctine phylogeny is pivotal to understanding the biological variability of non-pollinating fig wasps in the Old World. It is noteworthy that maritime Southeast Asia comprises thousands of tropical, segregated islands. The species richness across these habitats is consistently underestimated [26]. *F. hirta* is also distributed on some surrounding islands, such as Kalimantan. We may find more species if we identified the samples of non-pollinating fig wasps from more islands. Hence, additional sampling is necessary to establish a solid reference for further comparative studies, especially within mainland and maritime Southeast Asia.

## Figures and Tables

**Table 1:** Sampling sites for sycoryctine fig wasps associated with *Ficus hirta*.

Wasp Species	Country	Site	Latitude, Longitude
*Philotrypesis guangdongensis*	China	Gui	25.077, 110.306
		Huo	23.170, 113.373
		DHS	23.166, 112.543
		Xiang	22.424, 114.306
		Nan	22.787, 108.389
		Ding	19.697, 110.328
		Wan	18.795, 110.391
	Thailand	QMS	18.809, 98.914
		CH	12.774, 102.096
		Wu	14.443, 105.273
		HB	12.999, 108.230
		ST	7.467, 99.639
*P. yunnanensis*	China	Sand	25.984, 107.874
		XI	21.913, 101.264
	Thailand	QMS	18.809, 98.914
		Tai	18.894, 98.858
		CS	18.84, 99.47
*P. fujianensis*	China	Ning	26.664, 119.549
		Sha	26.419, 117.818
		Xia	24.742, 118.072
		Sui	26.476, 114.239
		Da	24.258, 116.806
*Sycoryctes javaensis*	Indonesia	CI	−6.566, 106.706
		JA	−6.368, 106.830
*Sycoscapter chinensis*	China	Gui	25.077, 110.306
		Huo	23.170, 113.373
		DHS	23.166, 112.543
		Xiang	22.424, 114.306
		Sand	25.984, 107.874
		Nan	22.787, 108.389
		Ding	19.697, 110.328
		Wan	18.795, 110.391
		XI	21.913, 101.264
*S. thaiensis*	Thailand	CH	12.774, 102.096
		Wu	14.443, 105.273
		Tai	18.894, 98.858
		CS	18.84, 99.47
		QMS	18.809, 98.914
*S. singaporensis*	Singapore	SNP	1.312, 103.816

## Data Availability

Not applicable.

## References

[1] Cook J.M., Rasplus J.-Y. (2003). Mutualists with attitude: Coevolving fig wasps and figs. Trends Ecol. Evol..

[2] Jousselin E., Van Noort S., Greeff J.M. (2004). Labile male morphology and intraspecific male polymorphism in the Philotrypesis fig wasps. Mol. Phylogenetics Evol..

[3] Hawkins B.A., Compton S.G. (1992). African fig wasp communities: Undersaturation and latitudinal gradients in species richness. J. Anim. Ecol..

[4] Van Noort S., Rasplus J. (1997). Revision of the otiteselline fig wasps (Hymenoptera: Chalcidoidea: Agaonidae), I: The Otitesella digitata species-group of the Afrotropical region, with a key to Afrotropical species of Otitesella Westwood. Afr. Entomol..

[5] Segar S.T., Lopez-Vaamonde C., Rasplus J.-Y., Cook J.M. (2012). The global phylogeny of the subfamily Sycoryctinae (Pteromalidae): Parasites of an obligate mutualism. Mol. Phylogenetics Evol..

[6] Weiblen G.D. (2002). How to be a fig wasp. Annu. Rev. Entomol..

[7] Jiang Z.F., Huang D.W., Chen L.L., Zhen W.Q., Fu Y.G., Peng Z.Q. (2006). Rampant host switching and multiple female body colour transitions in Philotrypesis (Hymenoptera: Chalcidoidea: Agaonidae). J. Evol. Biol..

[8] Wong D.-M., Bain A., Chou L.-S., Shiao S.-F. (2018). Description of two new species of fig wasps (Chalcidoidea: Pteromalidae: Sycoryctinae) associated with Ficus benguetensis. Taiwania.

[9] Pramanik A., Dey D. (2019). Two new fig wasp species of genus Sycoscapter Saunders, 1883 (Hymenoptera: Chalcidoidea: Pteromalidae) with a key to species of the genus from India. Taiwania.

[10] McLeish M.J., Beukman G., van Noort S., Wossler T.C. (2012). Host-plant species conservatism and ecology of a parasitoid fig wasp genus (Chalcidoidea; Sycoryctinae; Arachonia). PLoS ONE.

[11] Tzeng H.-Y., Tseng L.-J., Ou C.-H., Lu K.-C., Lu F.-Y., Chou L.-S. (2008). Confirmation of the parasitoid feeding habit in Sycoscapter, and their impact on pollinator abundance in Ficus formosana. Symbiosis.

[12] Kuttamathiathu J. (1959). The biology of Philotrypesis caricae (L.), parasite of Blastophaga psenes (L.) (Chalcidoidea: Parasitic Hymenoptera). Proc. Int. Congr. Zool.

[13] Segar S.T., Cook J.M. (2012). The dominant exploiters of the fig/pollinator mutualism vary across continents, but their costs fall consistently on the male reproductive function of figs. Ecol. Entomol..

[14] Deng X., Chen L., Tian E., Zhang D., Wattana T., Yu H., Kjellberg F., Segar S.T. (2021). Low host specificity and broad geographical ranges in a community of parasitic non-pollinating fig wasps (Sycoryctinae; Chalcidoidea). J. Anim. Ecol..

[15] Noyes J.S. (1982). Collecting and preserving chalcid wasps (Hymenoptera: Chalcidoidea). J. Nat. Hist..

[16] Kawada R., Buffington M.L. (2016). A scalable and modular dome illumination system for scientific microphotography on a budget. PLoS ONE.

[17] Gibson G., Huber J., Woolley J. (1997). Annotated Keys to the Genera of Nearctic Chalcidoidea (Hymenoptera).

[18] Yoder M.J., Mikó I., Seltmann K.C., Bertone M.A., Deans A.R. (2010). A gross anatomy ontology for Hymenoptera. PLoS ONE.

[19] Berg C., Corner E. (2005). Moraceae (Ficus).

[20] Yu H., Zhao N.-X., Chen Y.-Z., Deng Y., Yao J.-Y., Ye H.-G. (2006). Phenology and reproductive strategy of a common fig in Guangzhou. Bot. Stud..

[21] Chen Y., Compton S.G., Liu M., Chen X.Y. (2012). Fig trees at the northern limit of their range: The distributions of cryptic pollinators indicate multiple glacial refugia. Mol. Ecol..

[22] Bain A., Borges R.M., Chevallier M.-H., Vignes H., Kobmoo N., Peng Y., Cruaud A., Rasplus J.-Y., Kjellberg F., Hossaert-Mckey M. (2016). Geographic structuring into vicariant species-pairs in a wide-ranging, high-dispersal plant–insect mutualism: The case of Ficus racemosa and its pollinating wasps. Evol. Ecol..

[23] Rodriguez L.J., Bain A., Chou L.-S., Conchou L., Cruaud A., Gonzales R., Hossaert-McKey M., Rasplus J.-Y., Tzeng H.-Y., Kjellberg F. (2017). Diversification and spatial structuring in the mutualism between Ficus septica and its pollinating wasps in insular South East Asia. BMC Evol. Biol..

[24] Yu H., Tian E., Zheng L., Deng X., Cheng Y., Chen L., Wu W., Tanming W., Zhang D., Compton S.G. (2019). Multiple parapatric pollinators have radiated across a continental fig tree displaying clinal genetic variation. Mol. Ecol..

[25] Yu H., Liang D., Tian E., Zheng L., Kjellberg F. (2018). Plant geographic phenotypic variation drives diversification in its associated community of a phytophagous insect and its parasitoids. BMC Evol. Biol..

[26] Rodriguez L., Cruaud A., Rasplus J.-Y. (2020). Low sampling effort and high genetic isolation contribute to under-documented diversity in Philippine fig wasps. Philipp. J. Sci..

